# Supplementary figures and images for: Clinical implications of APOBEC3A and 3B expression in patients with breast cancer
Source: PLoS One. 2020 Mar 16;15(3):e0230261. doi: 10.1371/journal.pone.0230261 (PMC7075570; doi:10.1371/journal.pone.0230261)

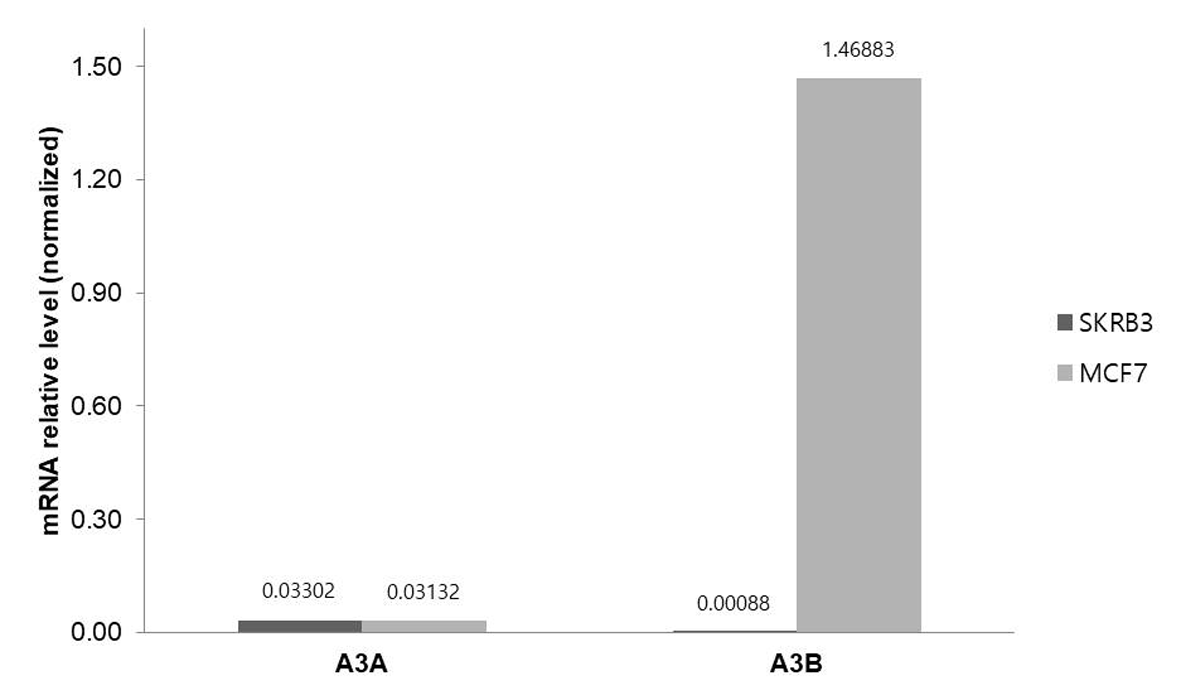

Supplement: S1 Fig — (TIF) [file pone.0230261.s001.tif]
