# Supplementary material for: Clinical implications of APOBEC3A and 3B expression in patients with breast cancer
Source: PLoS One. 2020 Mar 16;15(3):e0230261. doi: 10.1371/journal.pone.0230261 (PMC7075570; doi:10.1371/journal.pone.0230261)
Supplement: Attachment — Submitted filename: APOBEC_Response.docx [file pone.0230261.s004.docx]

**Clinical implications of APOBEC3A and 3B expression in patients with breast cancer**

Dear Editor,

We’re very grateful for the constructive comments of the reviewers. It is clear that comments make our paper better. We revised our manuscript in response to reviewers’ comment. We have highlighted the major changes with a blue color. We hope that the revision satisfies reviewers’ requirement. Thank you very much.

Reviewer 1

1) Because the authors are focused an Asian populations (Korean), where an A3A-B deletion allele is common, they should also consider A3H haplotypes which Starrett and coworkers implicated in causing APOBEC signature mutations in the absence of A3B (PMID 27650891). In other words, A3H could also be clinically relevant.

🡪 We appreciate your good comments. New findings on APOBEC mutation signature in cancer have been reported in recent years. We planned to investigate the most studied APOBEC3A and 3B in our patients, because there was not much data on other APOBEC family including APOBEC3H in cancer. We added the A3H haplotype you mentioned as a limitation of our study in discussion.

2) The RTqPCR assays for A3A and A3B may not be specific due to extremely high homology between these genes. What tests have the authors done to validate these assays and ensure specificity?
🡪 We agree with reviewer’s opinion. We confirmed that using Primer-BLAST-NCBI. We attached the captured image of the results.

3) Lines 202-207 – what are the units for A3A and A3B mRNA levels? This is unclear. All raw values should be provided in a supporting table (ie. not just medians).

🡪 Thank you for your comments. The relative mRNA expression level was normalized to the β-actin gene expression, and then expressed as thelog2 fold change of the delta-delta Ct values. We added it in Method. We submit all raw values as supplement file (S1).

4) It is hard to believe that there are no patients completely lacking A3B (ie. homozygous null) given the 77/(138x2) allele frequency of the deletion in this population. Can the deletion allele be verified using an independent assay such as with different PCR primers and sequencing?
🡪 Yes, the deletion allele was verified using different PCR primers. We rechecked for heterozygous deletion/ insertion using insertion primer in samples that showed homozygous deletion in genotyping. (Reference: Kidd JM et al. Population stratification of a common APOBEC gene deletion polymorphism. PLoS Genet 3(4): e63)

5) The rationale for correlating serum sPD-L1 and tumor A3A and A3B mRNA levels is not explained. This analysis makes no sense at all.
🡪 Thank you for your comment. APOBEC is well known as an endogenous mutagen in various cancers. High tumor mutational burden and APOBEC mutagenesis signature are associated with immune signatures and with increased expression of immune-related genes. Therefore, we wanted to evaluate the relationships between APOBEC mRNA levels and serum levels of sPD-L1 and IFN-γ as immune-related markers. Obviously, there are better ways to evaluate cancer immunogenicity including immune cell infiltration. Unfortunately, we were not able to evaluate it properly. We described this as a limitation of our research in the discussion.

Minor

- Line 100 should also cite PMID:23389445, which is the first paper to implicate A3B in cancer.

🡪 We added that paper according to your comment. Thank you.
- Line 161 – dNTPs

🡪 We revised it. Thank you.

Reviewer 2

1. How were ER expression and Her2 expression measured and what were the cut-offs for being called ER positive and Her2 positive? What proportion of Her2 patients are also ER positive?

🡪 Thank you for your comments. We added definitions of hormone receptor positivity and HER2 positivity in method. Among 49 patients with HER2-positive breast cancer, 34 patients were also HR-positive. We added it in result.

2. Does the RTPCR for A3A also amplify the deletion allele?
🡪 With reference to other papers measuring A3A mRNA levels, we used the A3A primer that was amplified from both the normal A3A allele and the deletion allele.

3. Are the mRNA levels reported in the tables deltadeltaCT values, this is not completely clear from the methods?

🡪 Thank you for your comments. The relative mRNA expression level was normalized to the β-actin gene expression, and then expressed as the log2 fold change of the delta-delta Ct values. We added it in Method.

4. What is meant by “direct” sequencing – was this Sanger sequencing? Why was cDNA analysed and not genomic DNA? Furthermore, the exact mutations detected for each sample should be tabulated and provided as supplementary data.

🡪 To evaluate mutations in exons, cDNA was amplified by PCR with each primer of TP53 and PIK3CA, and then we did Sanger sequencing for the products. Finally, we analyzed the mutations by comparing the sequencing results with reference sequence. We submit all raw values as supplement file (S2).

5. Which statistical tests were used to generate the p values in the tables? Due to the large interquartile range I’m surprised at some of the p values. I would like to note however that as I am not familiar with all the statistical tests outlined in the methods and am unable to say whether they are the most appropriate statistical test for this data. In addition, without the full data being available (the authors only provide summary statistics) it is hard to test the veracity of the statistics.
🡪 To compare the differences of continuous variables between two or more groups, we performed Mann-Whitney U test and Kruskal-Wallis test as nonparametric tests because our data isn’t normally distributed. We added the statistical methods below the table and figure.

6. What two variables are being compared to generate the statistics in fig 1? My understanding is that the K-W test that was used can demonstrate that one sample significantly dominates the other samples in the analysis, but it does not identify which sample is the dominant one and this needs to be determined using a post-hoc test?
🡪 We appreciate your good comment. Dunn-Bonferroni post hoc testing is carried out on each pair of groups after a Kruskal-Wallis test. We added detailed information and results about this.

B. The high prevalence of the deletion phenotype in this cohort (56%) makes the mRNA analysis of A3A problematic. I presume that the A3A RTPCR used will amplify mRNA from both the normal A3A allele and the deletion allele, while the A3B RTPCR would only amplify mRNA from the normal A3B allele? If this is not the case this should be clarified in the methods. If it does amplify both alleles this means that for a large number of patients the read out from the A3A assay will be measuring a combination of A3A and mutant gene expression. Thus, from the authors analysis they cannot categorically state the associations are due to A3A mRNA levels, rather than the deletion allele mRNA. While the deletion allele is identical in coding sequence the change in the 3’ UTR could change the stability of the mRNA and also the rate of translation of mRNA to protein. As the mutant allele has been associated with increased risk of breast cancer by a number of other groups the prevalence of this allele is important to the understanding of A3A biology (there is a meta-analysis of this in this publication: 10.18632/oncotarget.19400). This issue could affect a large number of the subsequent analyses that compare other clinical parameters to supposed A3A mRNA levels. Ideally the investigators would redo this analysis with an assay which can separately measure the mutant allele and normal allele.
🡪 We appreciate your comment and understand your suggestion. We used the primer mentioned in previous studies. It measured the A3A mRNA levels from both deletion allele and non-deletion allele. As you mentioned, there may be differences between A3A mRNA from deletion allele and A3A mRNA from non-deletion allele. Some study reported that APOBEC3B expression was significantly lower in A3B deletion carriers, but APOBEC3A expression was not observed to be significantly higher in A3B deletion carriers. Some study mentioned that A3A mRNA from deletion allele was more stable, resulting in higher intracellular A3A levels. We think that further studies are needed to clarify the effect of A3B deletion allele on A3A mRNA levels and the best method to measure A3A mRNA levels in A3B deletion carriers. We added this in discussion. Thank you for your important advice.

C. The rationale for studying the link between A3A and A3B expression levels and immune related parameters such as serum IFN-gamma and sPDL1 seems tenuous. In this cohort they have not shown that the level of A3A associates with mutational burden or immune cell infiltration into the tumour. Furthermore, as they were unable to measure IFN-gamma in a large number of samples and the association of sPDL1 with A3A is marginal (R2=0.03) I think this data is not very useful/informative. Particularly as the association may or may not be confounded by the relative abundance of the deletion allele.
🡪 Thank you for your comment. We agree with your opinion. We describe this as a limitation of our research in the discussion.

D. As the investigators show that the levels of A3B associate with the TNBC subtype then subsequent associations of A3B with clinical parameters need to have the caveat that the association may be due to differences in breast cancer subtype proportions within the groups studied rather than A3B directly.
🡪 We understand your concerns. However, correlation with clinical parameters showed more apparent in A3A than in A3B. Actually, there is no big difference in A3A mRNA levels between subtypes. Therefore, we think that it’s hard to say that associations of A3B with clinical parameters are only caused by differences in breast cancer subtype proportions.

E. I do not think that the survival analyses are informative and should be removed. As they only finished collecting samples from this cohort in December 2016 there is insufficient follow-up time to draw conclusions about survival. If they do want to include survival data then the Kaplan-Meier curves need to be included, and these should also state the numbers of censored events over time. In addition, it should be noted that the survival data could also be influenced by other factors such as that the population analysed contains all the different subtypes of breast cancer that would each have been treated differently (hormone therapy for ER pos, Herceptin for Her2, and chemo for TNBC). Although the numbers would be quite small they could do separate analyses for each subtype of breast cancer.
🡪 We agreed with your opinion. Survival analyses have some limitations due to small number of patients and short follow-up time. We decided to remove the survival data according to your recommendation.

Minor points:
This cohort seems unusual with only 40% luminal breast cancer patients (most cohorts have >70% luminal subtype), the authors should comment on why this is the case. Is this normal for a Korean population or did the selection criteria skew the relative abundance of luminal patients?

🡪 We agreed with your opinion. Luminal subtype accounts for about 60-70% of all patients with breast cancer. Based on the clinicopathological data, we chose samples with similar proportions for each subtype to prevent the number of one subtype from becoming too small. We added this comments in method. Thank you.

The authors should also clarify whether the deletion allele is associated with any particular subtype of breast cancer (this is not completely clear from table 2 as they do not include breast cancer subtypes).

🡪 We described that in result (Line 241-242):There was no difference in the frequency of A3B deletion according to breast cancer subtypes.
