# Supplementary material for: Clinical implications of APOBEC3A and 3B expression in patients with breast cancer
Source: PLoS One. 2020 Mar 16;15(3):e0230261. doi: 10.1371/journal.pone.0230261 (PMC7075570; doi:10.1371/journal.pone.0230261)
Supplement: Attachment — Submitted filename: 2ndRevision_response.docx [file pone.0230261.s005.docx]

**Clinical implications of APOBEC3A and 3B expression in patients with breast cancer**

Dear Editor,

We once again appreciate good comments of the reviewers and it is definitely helpful. We revised our manuscript in response to reviewers’ comments. We have highlighted the major changes with a blue color. We hope that the revision satisfies reviewers’ requirement. Thank you very much.

Reviewer 1

1) The authors have not yet addressed my most important comments. Just because they are using published PCR assays does not mean they are excused from doing proper controls for assay specificity (prior work could be incorrect). For instance, both RTqPCR and PCR assay specificity could be validated using breast cancer cell lines with known APOBEC genotypes. SKBR3, for instance, is A3B null (PMID 23389445). MCF10A is wildtype (as far as anyone knows) and A3B is inducible with PKC agonists and A3A with interferon-alpha (PMID 26420215).

🡪 We appreciate your good comment. According to your recommendation, we checked the A3A and A3B mRNA expression levels in SKBR3 and MCF7 breast cancer cell lines. We confirmed that A3B mRNA level in SKBR3 (A3B null type) cells was relatively very low (undetectable level). We submited the result as a supporting figure (S1 Fig). We described that in method.

Reviewer 2

Major points:
1) In the discussion they state they use matched adjacent normal tissue in their analyses but in the results section they state only 10 normal tissue samples were used and from the supplementary table 1 it appears they have used an average normal value that is not matched to the patient tumour samples. This needs to be clarified. In addition, did they include a similar distribution of mutant allele carriers in the normal tissue as in the tumour samples? Does the deletion allele affect expression of A3A and A3B in normal tissue? If this could be a confounding factor it should be discussed.

🡪 Thank you for your comment. First, to make sure that A3A and A3B mRNA levels are increased in tumor tissues rather than normal breast tissues, we got the mRNA samples from 138 breast tumor tissues and 10 adjacent normal breast tissues of 138 patients, and compared the mRNA levels between tumor tissues and normal tissues. Second, in the case of genotyping assay of germline APOBEC3A/B deletion polymorphism, it is not a somatic mutation but a germline mutation. Therefore, we got the genomic DNA from 138 matched normal breast tissues, not tumor tissues. We’re sorry for causing confusion. To make this clear, we added the number of cases in each method, results and discussion.

2) In both the results and discussion, it is stated that A3A and A3B are higher in younger patients, however, the results that are in table 1 show the opposite, with higher median levels of both A3A and A3B in the older (>52) patients. This needs to be clarified and corrected.
🡪 Thank you for finding our mistake. We confirmed that the result in Table was correct. We revised the sentence in results and discussion.

Minor points:
1) The full breast cancer subtype data and associations with A3A, A3B and the mutation allele should be included in Tables 1 and 2 for completeness.

🡪 We appreciate your good comment. We added and revised the breast cancer subtype data in Table 1 and 2 according to your recommendation.

2) Were outliers included or excluded in the generation of the line of best fit for the R-squared analysis of sPDL1 and A3A levels? While there is a significant association between sPDL1 and A3A levels the R squared value is very low, this should be noted in the results or discussion.

🡪 We appreciate your good comment. We included all the values in correlation analysis. We changed coefficient of determination (r^2^) to correlation coefficient (r). The data has showed that there is a significant weak positive correlation (r = 0.264, p = 0.017). We mentioned this in results and discussion.

3) In the introduction it is stated that previous studies have shown that the Apobec mutation profile is highest in the Her2 subtype, this should be commented on in relation to the results from this study in the discussion.

🡪 Thank you for your comment. We added this in the discussion according to your recommendation.

4) A number of times it is stated that A3B levels are significantly higher in TNBC, this should be qualified with the fact that this is only in comparison to HR positive cancers not Her2 positive.

🡪 Thank you for your comment. We agreed with your opinion. We revised the sentence in results and discussion.

5) The number of Her2 positive patients in Table 2 equals 48 whereas the text states there were 49 Her2 patients.

🡪 Thank you for your comment. We revised the breast cancer subtype data according to your previous recommendation (No.1).

6) The initial manuscript was written with good English however a number of the revisions/edits added to the manuscript following the initial review need to be checked for grammar.

 🡪 We agree with your opinion. We will correct the final version again via professional editing service web site (Online English -http://www.oleng.com.au/).
